# Optical Myography-Based Sensing Methodology of Application of Random Loads to Muscles during Hand-Gripping Training

**DOI:** 10.3390/s24041108

**Published:** 2024-02-08

**Authors:** Tamon Miyake, Tomohito Minakuchi, Suguru Sato, Chihiro Okubo, Dai Yanagihara, Emi Tamaki

**Affiliations:** 1 H2L Inc., Tokyo 106-0032, Japanemi@h2l.jp (E.T.); 2 Future Robotics Organization, Waseda University, Tokyo 169-8050, Japan; 3 Graduate School of Engineering and Science, University of the Ryukyus, Okinawa 903-0129, Japan; 4 Department of Life Sciences, Graduate School of Arts and Sciences, The University of Tokyo, Tokyo 153-8902, Japan; dai-y@idaten.c.u-tokyo.ac.jp; 5 Faculty of Engineering, University of the Ryukyus, Okinawa 903-0129, Japan

**Keywords:** optical myography, muscle deformation, hand gripping

## Abstract

Hand-gripping training is important for improving the fundamental functions of human physical activity. Bernstein’s idea of “repetition without repetition” suggests that motor control function should be trained under changing states. The randomness level of load should be visualized for self-administered screening when repeating various training tasks under changing states. This study aims to develop a sensing methodology of random loads applied to both the agonist and antagonist skeletal muscles when performing physical tasks. We assumed that the time-variability and periodicity of the applied load appear in the time-series feature of muscle deformation data. In the experiment, 14 participants conducted the gripping tasks with a gripper, ball, balloon, Palm clenching, and paper. Crumpling pieces of paper (paper exercise) involves randomness because the resistance force of the paper changes depending on the shape and layers of the paper. Optical myography during gripping tasks was measured, and time-series features were analyzed. As a result, our system could detect the random movement of muscles during training.

## 1. Introduction

Hand-gripping is one of the fundamental functions of human physical activity. In particular, hand-gripping strength is important in a number of sports, such as golf, tennis, hockey, and baseball [1]. The hand is the main part of the body involved in making physical contact with an object. Sport-specific hand actions require a power grip or precision grip [2]. The hand-grip strength plays an important role in throwing and swinging a racket, bat, stick, or club. Hand-grip strength of elites and successful athletes was greater than that of sub-elites and less successful counterparts [3,4]. In addition, the timing and sequencing of hand-gripping force applied to an object during sport-specific movement patterns, such as swinging and throwing an object, are also important factors [5,6].

The hand-gripping force is exerted as a total activation of both the agonist muscles (forearm flexor muscles) and the antagonist muscles (forearm extensor muscles) [7]. Isometric hand-gripping training was applied to improve the hand-gripping force [8,9]. Even when only forearm extensor muscles are strengthened based on isometric wrist extension training, the hand-gripping force increases [10]. Isokinetic exercise on skeletal muscle with a spring-loaded hand-gripper had a greater training effect than isometric exercise [11]. These isometric and isokinetic exercises are methods of applying load to skeletal muscles and improving skeletal muscle force.

As known as Bernstein’s degrees-of-freedom problem in motor control, our nervous system needs to reduce redundancy, which is inherent in our body for efficient movement [12]. Multiple skeletal muscles need to be activated corresponding to an environment for physical activity. A sophisticated sensorimotor function of temporal and spatial control is required for high-level performance [13,14]. Sophisticated sensorimotor movement involves variability of movement trajectory, such as the golf swing movement [15]. This phenomenon implies that people adjust their motion corresponding to slight changes in the environment or situation. Motor adaptation to an environment is the acquisition of predictive adjustment as feedforward control [16]. A large number of training of motor tasks leads to improvement in motor adaptation ability [17].

Bernstein’s idea of “repetition without repetition” suggests that motor control function should be trained under changing state [12]. The perturbation-based training paradigm is one type of training where the environment changes randomly to promote motor adaptation of neuromuscular function to the changeable environment [18,19,20,21]. For example, perturbation leads to more precise control of toe movement based on more effective covariation of redundant lower limb segments during walking [19]. A swinging load applied during bench press training increases the number of activated skeletal muscles or increases the activity level of the skeletal muscles [22,23]. Control of the forearm skeletal muscles during wrist flexion and extension perturbations involves greater co-contraction of agonist and antagonist skeletal muscles of the forearm [24,25], which results in greater wrist joint stiffness, which is a measure of the rigidity of the wrist joint [26]. The perturbation devices are specified devices to apply random stimulation to the hand muscles [24,25].

It is important to monitor the time-series features of the load to enable trainees to conduct self-administered screening during daily sports training. In particular, the randomness level of load should be visualized for self-administered screening when repeating various training tasks under changing states. Previous studies evaluated the strength or power of the load using muscle activity [27,28], accelerations [29,30], and flexion angle [31]. However, the quick and easy methodology for evaluating the time-variability and periodicity of the applied load to the skeletal muscles has not been established.

## 2. Related Works

There are some biomarkers to observe muscle activity: electromyography (EMG), force myography (FMG), and optical myography (OMG). EMG is a myographic method based on electrical signals from the brain to the muscles [32]. EMG is a pulse wave-type signal, and some processes, such as rectification and smoothing, are needed to derive the applied loads to the skeletal muscles. In addition, time-variability and periodical analysis for EMG was conducted for analyzing muscle fatigue [33,34,35]. EMG changes due to the condition change of the skin, such as sweating. In addition, the EMG signal can be affected easily by small displacements of the electrode position.

FMG and OMG methods estimate the deformation of skin or skeleton muscles. FMG is a myographic method based on interface force information between sensors and a body [36]. FMG is required to attach sensors by keeping applying certain pressure to the skeletal muscles, and then might cause discomfort after a prolonged time of use. OMG is a myographic method based on optical information [37]. In particular, a near-infrared sensor is used to monitor the skeletal muscle activities [38,39,40,41,42] A near-infrared light is strongly scattered by biological tissue when it propagates through the tissues [43], which has a potential that the reflectance information includes relatively deeper muscle information. The reflectance of the near-infrared light changes due to muscle oxygenation activity involved by muscle deformation because hemoglobin and myoglobin absorb the near-infrared light [44,45]. In addition, the reflectance of the near-infrared light also changes even with skin deformation [46]. Due to the complexity of reflectance of the near-infrared light, whether near-infrared-based OMG can observe the time-variability and periodicity of the applied load to the skeletal muscles is unclear.

## 3. Objective

We aim to establish the methodology to identify the time-variability and periodicity of the loads to skeletal muscles during sports training. The objective of this study is to develop a sensing methodology of random loads applied to both the agonist and antagonist skeletal muscles while performing physical tasks. The state of the amount of load on the agonist and antagonist skeletal muscles (flexor and extensor muscles of the hand and finger joints) was analyzed with several training techniques. To our knowledge, this is the first study to examine the temporal pattern of skeletal muscle deformation during a variety of hand-gripping training.

We assumed that the time-series feature of the applied load appears in the time-series feature of muscle deformation data. Strength, variability, and periodicity of muscle deformation data were investigated to evaluate whether the system could detect the random load applied to the skeletal muscles or not.

## 4. Materials and Methods

### 4.1. System

**Figure 1:**
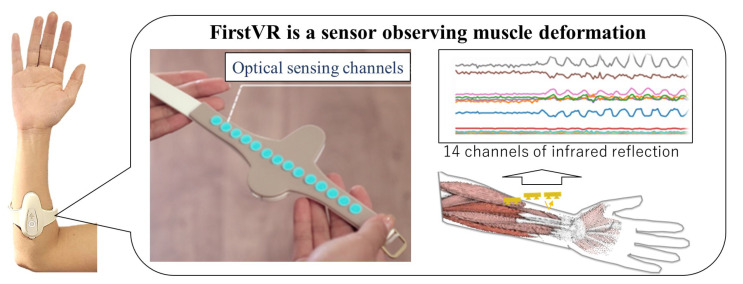
FirstVR, a muscle deformation sensor with 14-channels based on near-infrared OMG.

Because OMG is simpler, more comfortable, intuitive, and inexpensive, we selected OMG as the biomarker for the measurement of muscle activity. The signals from the 14 channels of a FirstVR (H2L Inc., Tokyo, Japan) were obtained. The FirstVR was used because this is an OMG sensor that can obtain information on the forearm skeletal muscle deformation, which enables estimation of the fingertip force [47]. As shown in Figure 1, the FirstVR is a band-type sensor based on near-infrared optical sensing consisting of infra-light emitting and infra-light receiving. When skeletal muscles are activated, the skeletal muscle deforms by muscle contraction. The reading value in each channel of the FirstVR changes when the skeletal muscle or skin deforms. The attachment cover was modified by replacing the Urethane sponge from the original FirstVR to clearly observe muscle deformation. The reading values were sent to a computer from FirstVR through Bluetooth Low Energy. The sampling frequency of the data recording was approximately 9 Hz. The sensor is mounted approximately one-forth of the forearm length from the elbow.

We considered the indexes of the feature of the time series data of the muscle deformation. First, the SD was derived because the muscle state varies when a training task applies a random load. Next, the mean was analyzed to investigate whether the muscles were loaded or not. Third, the autocorrelation coefficient was examined to analyze the periodicity of the data. The autocorrelation coefficient is the correlation between the original data and the time-shifted data. If there is periodicity in the time series data, the autocorrelation coefficient will be higher if the shifted time (frame) is a multiple of the period of the data. A low autocorrelation coefficient indicates that the variation pattern of the data are random. To compare the autocorrelation coefficient among training tasks, the maximum value of the local maxima in the absolute value of the autocorrelation coefficient was extracted.

**Figure 2:**
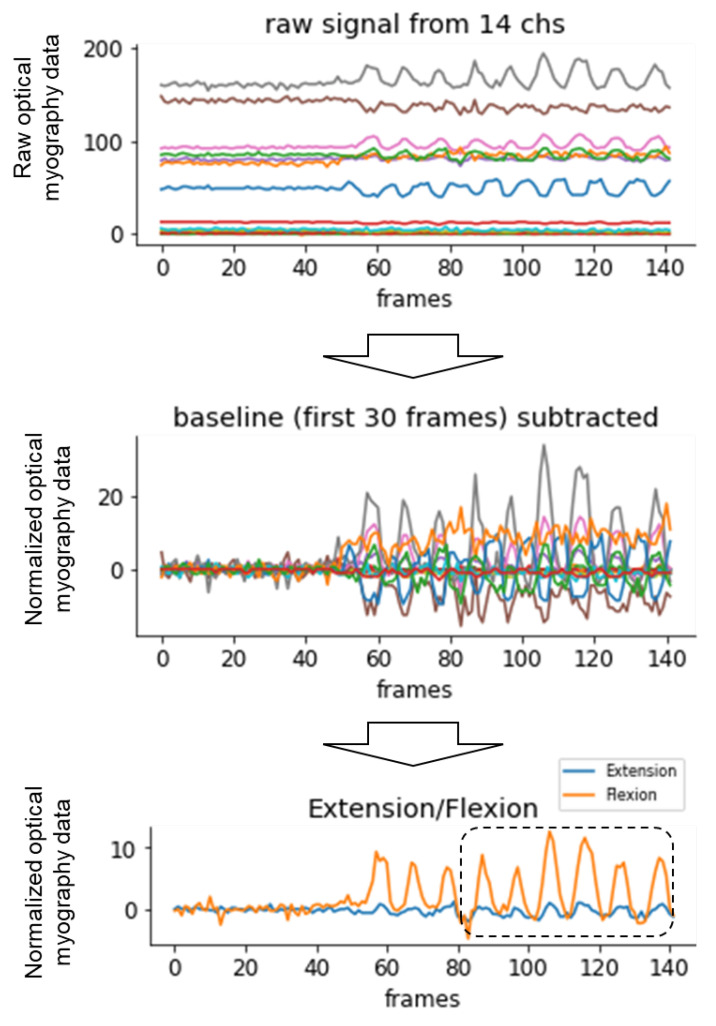
Data processing flow.The recording data were normalized by subtracting the baseline. The 14-channel signals were converted to the feature values representing the skeletal muscle deformation of the flexor and extensor muscles. In total, 80–140 frames were extracted to analyze the data.

Figure 2 indicates the data processing flow. First, of all, the recording data were normalized by subtracting the baseline (the averaged signal for the first 30 frames during relaxing) to set the reading value in the relaxed muscle state as zero in each channel. The 14-channel signals were converted to the feature values representing the skeletal muscle deformation of the flexor and extensor muscles. Because the timing of the start of the gripping varies among participants, data in the early frames were excluded from the analysis. In total, 60 frames (80–140 frames in Figure 2) were extracted in each task for each participant to analyze the data.

**Figure 3:**
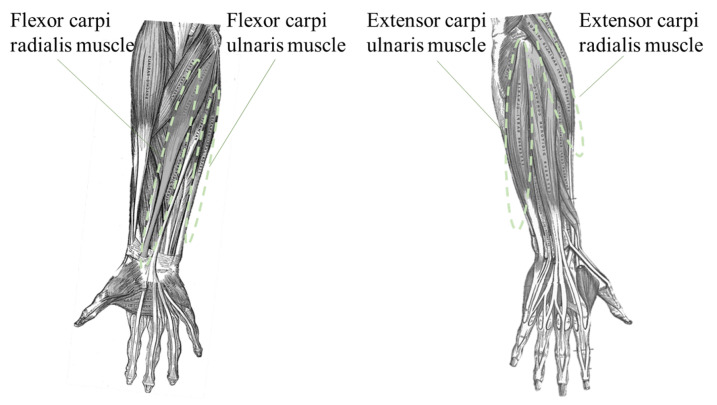
Sensed muscles by OMG. The observed flexor muscles are flexor carpi ulnaris muscle and flexor carpi radialis muscle. The observed extensor muscles are extensor carpi ulnaris muscle and extensor carpi radialis muscle.

Figure 3 shows the observed muscles by FirstVR in the experiment. The more the distances of the channels are, the lower the effect of the near-infrared emitter on the receiver is [42]. The observed flexor muscles are flexor carpi ulnaris muscle and flexor carpi radialis muscle. The observed extensor muscles are extensor carpi ulnaris muscle and extensor carpi radialis muscle. The channels named No. 5, No. 7, No. 8, and No. 10 overlapped the region of the flexor muscles. The channels named No. 1, No. 2, No. 4, No. 13, and No. 14 overlapped the region of extensor muscles. The average of the signals in each channel combination (the combination of No. 5, No. 7, No. 8, and No. 10, and the combination of No. 1, No. 2, No. 4, No. 13, and No. 14 combination) was derived as the feature value. The maximum value of the local maxima in the absolute value of the autocorrelation coefficient was derived for each time-series muscle deformation data (the averages of the channels) as the index of randomness of the applied load to the skeletal muscles. When attaching the FirstVR, we made a simple check by actually flexing and extending the wrist joint and visually checking the graph. The sensor attachment position was adjusted to set the above channels for measuring the target groups of muscles as much as possible.

### 4.2. Experimental Procedures

Fourteen healthy young participants (4 females and 10 males, aged 22–31 years) were recruited for the experiment. The number of participants was designed to provide an estimated statistical power greater than 0.8. We explained the content of the experiment to all participants. All experimental tasks were carried out with their consent (in writing). We also explained how participants could interrupt the experiment, so that they could stop the experiment at any time. We did not explain the purpose of the experiment. This experiment was approved by the Japanese Society for Wellbeing Science and Assistive Technology (No. 22-13).

**Figure 4:**
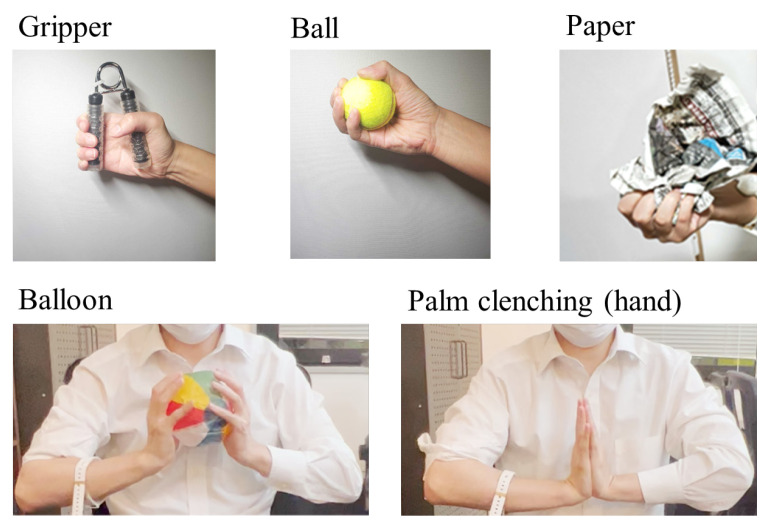
Training types in the experiment. Gripper: grip and release 25 kg hand grippers; ball: keep gripping a ball; palm clenching: keep clenching the hand; balloon: keep gripping a balloon; and Paper: crumple a piece of newspaper.

As shown in Figure 4, the participants conducted the following five training tasks.

- Gripper: grip and release a 25 kg hand-gripper approximately 10 times for 10 s.
- Ball: hold a tennis ball and keep exerting gripping force for 10 s.
- Palm clenching (hand): keep clenching the hand in front of the chest for 10 s.
- Balloon: hold a paper balloon in front of the chest for 10 s while applying force to the arms so as not to crush the balloon.
- Paper exercise: Crumple pieces of newspaper for 10 s. The number of paper layers was two.

The gripper and paper tasks were isokinetic exercises, and the other tasks were isometric exercises. The gripper task and the ball task were selected as the most typical hand-gripping training, where the human moves the skeletal muscles periodically. The resistance strength of the gripper was set to be approximately 50–55% and approximately 80–90% of means of the maximum gripping strength in male and female young populations, respectively [48]. The mean of the correlation coefficient between the peak value of the muscle deformation and peak number was approximately 0.25 during the gripper task. In addition, crumpling pieces of paper (paper exercise) involves randomness because the resistance force of the paper changes depending on the shape and layers of the paper.

The total duration of each task was 15 s. For the first 5 s, the forearm orientation was the same as in the task to reduce the effect of skin deformation. In addition, the wrist angle is 0 degrees to the forearm for the calibration data acquisition to keep skeletal muscles relaxed. After the first 5 s, training motion, such as gripping, started for 10 s. The interval was set only when the participants required this due to feeling fatigued.

### 4.3. Data Analysis

The OMG signals of the FirstVR were processed for analyzing muscle exertion of the flexor and extensor muscles. The standard deviations (SDs), means, and autocorrelation coefficients of the OMG signals, when each participant was engaging in the training, were compared between the training types for each muscle.

The distribution of the SDs, means, and autocorrelation coefficients did not pass the normality test, thus we used nonparametric Kruskal–Wallis test to confirm that these parameters of muscle deformation were significantly different among the training types. We also used a Dwass, Steel, Critchlow and Fligner all-pairs comparison test (DSCF test) for the pairwise comparison between two different training types. Statistical tests were performed using SciPy (version 1.3.0, for Kruskal–Wallis test) and scikit_posthocs (version 0.7.0, for DSCF test). A *p*-value of 0.05 was set as the significance level.

## 5. Results

**Figure 5:**
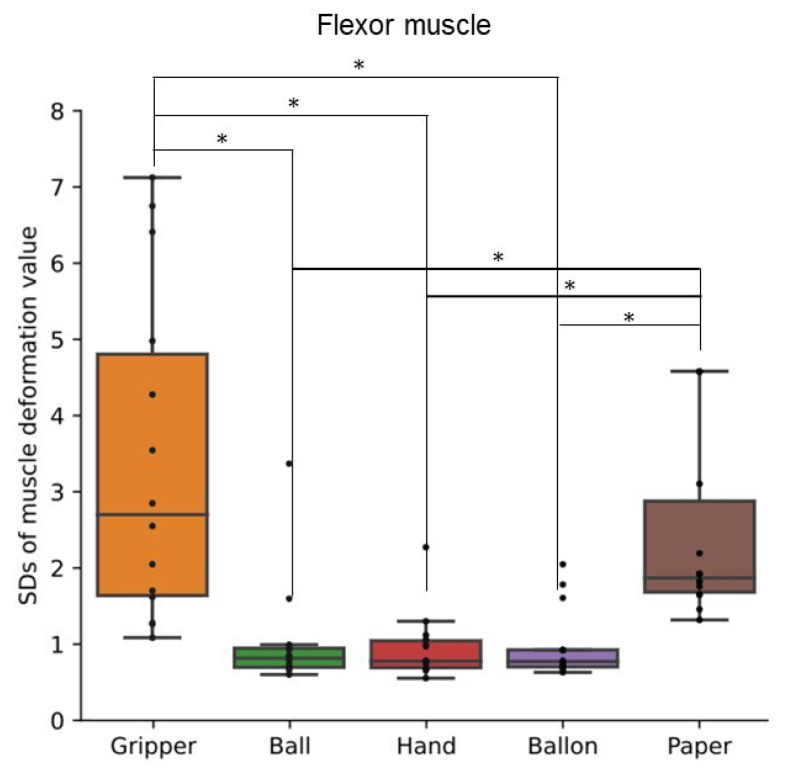
Standard deviations of muscle deformation data for flexor muscles. Box plots of the standard deviations (SDs) of muscle deformation data for all participants’ flexor muscles. The training types were gripping–releasing of a gripper (gripper), holding of a ball (ball), palm clenching (hand), holding of a balloon (balloon), and crumpling of newspaper (paper). the symbol * means the significant difference.

**Figure 6:**
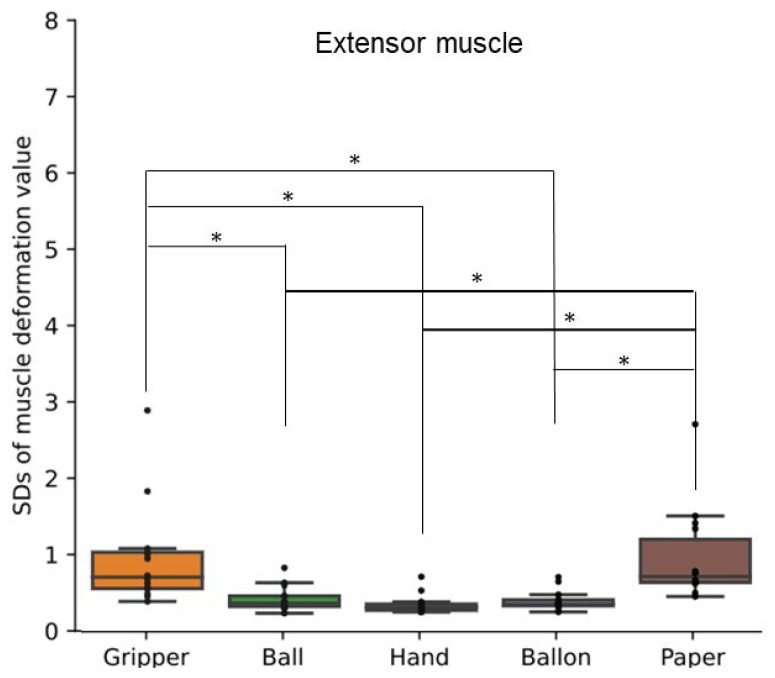
Standard deviations of muscle deformation data for extensor muscles. Box plots of the standard deviations (SDs) of muscle deformation data for all participants’ extensor muscles. The training types were gripping-releasing of a gripper (gripper), holding of a ball (ball), palm clenching (hand), holding of a balloon (balloon), and crumpling of newspaper (paper). The symbol * means the significant difference.

Figure 5 shows a box plot of SDs of muscle deformation data for all participants’ flexor muscles. Figure 6 shows a box plot of SDs of muscle deformation data for all participants’ extensor muscles. The SD indicates how much muscle deformation varies over time. The SD of the 25 kg gripper task was significantly different from the SDs of the ball, balloon, and hand (palm clenching) tasks. The SD of the paper task was not significantly different from the gripper task, but higher significantly than the SDs of the other tasks. The SDs derived by our processing of near-infrared signals detected fluctuation of the applied load to the forearm muscles over time for the gripper and paper tasks.

**Figure 7:**
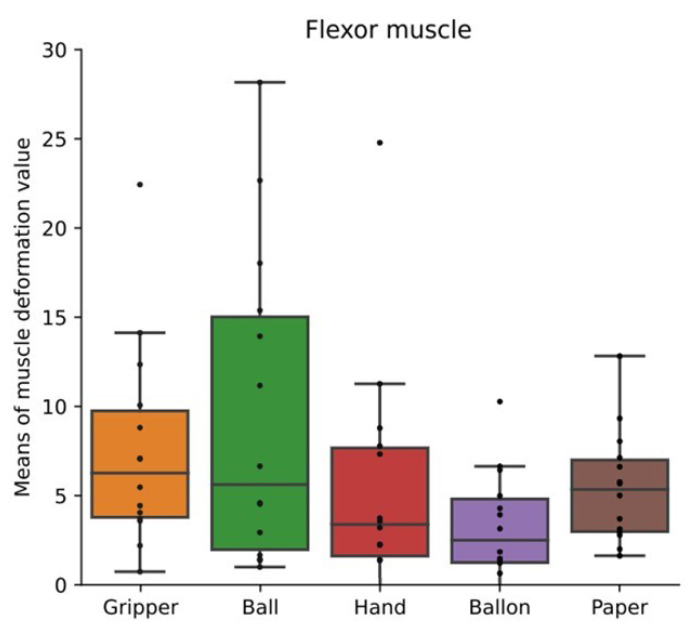
Mean values of muscle deformation flexor muscles. Box plots of mean values of muscle deformation data of all participants’ flexor muscles. Box plots of the maximum value of the local maxima in the absolute value of the autocorrelation coefficient for flexor and extensor muscles. The training types were gripping-releasing of a gripper (gripper), holding of a ball (ball), palm clenching (hand), holding of a balloon (balloon), and crumpling of newspaper (paper).

**Figure 8:**
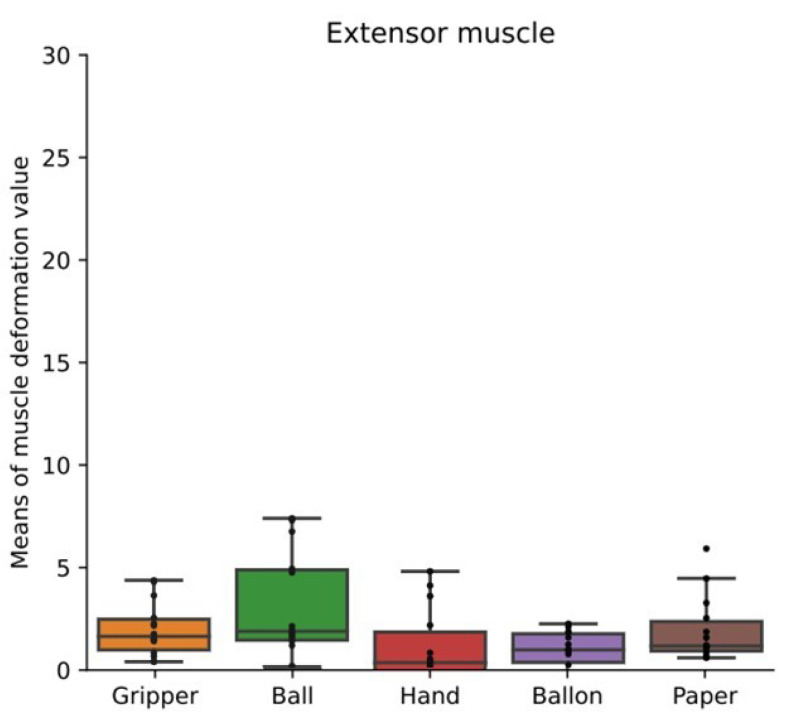
Mean values of muscle deformation data for extensor muscles. Box plots of mean values of muscle deformation for all participants’ extensor muscles. The training types were gripping-releasing of a gripper (gripper), holding of a ball (ball), palm clenching (hand), holding of a balloon (balloon), and crumpling of newspaper (paper).

Figure 7 shows box plots of mean values of muscle deformation data for all participants’ flexor muscles. Figure 8 shows box plots of mean values of muscle deformation for all participants’ extensor muscles. There was no significant difference among all the tasks. However, the mean value in the ball task, which is the isometric hand-gripping task, tended to be higher for some participants. The means could not detect any difference of the variability and periodicity of the applied load.

**Figure 9:**
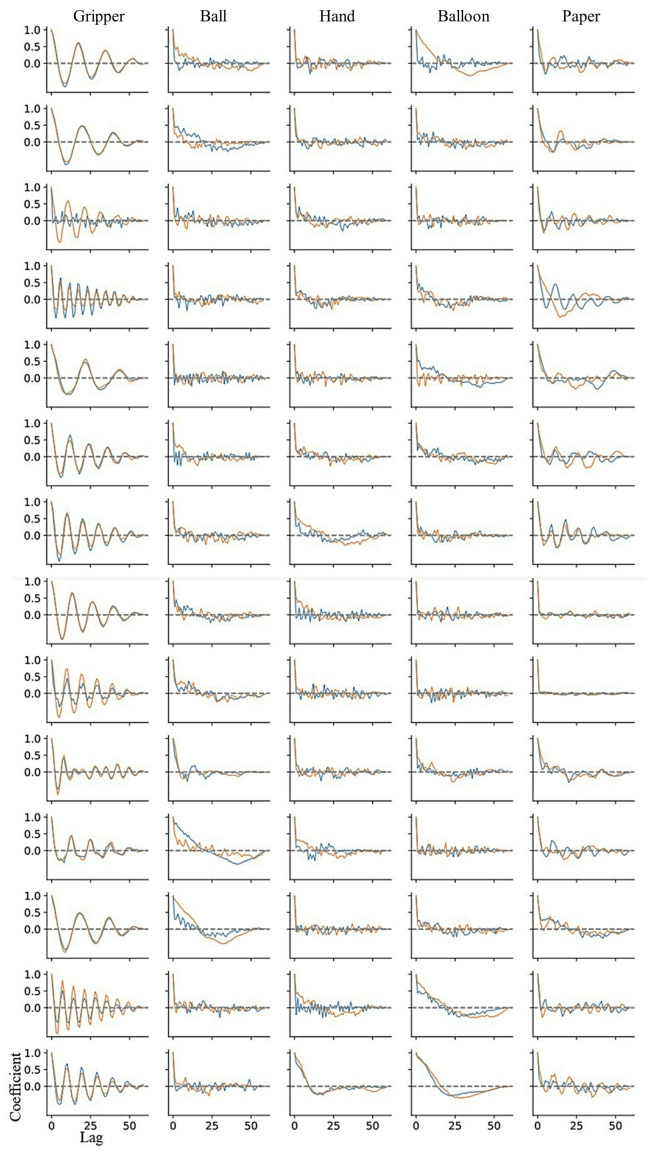
Autocorrelation coefficient in all the tasks for all participants. The graph is the correlogram, that is, the relationship between the autocorrelation coefficient value (vertical axis) and the shifted lag of the data (horizontal axis). The orange line indicates flexor muscles and the blue line indicates extensor muscles.

**Figure 10:**
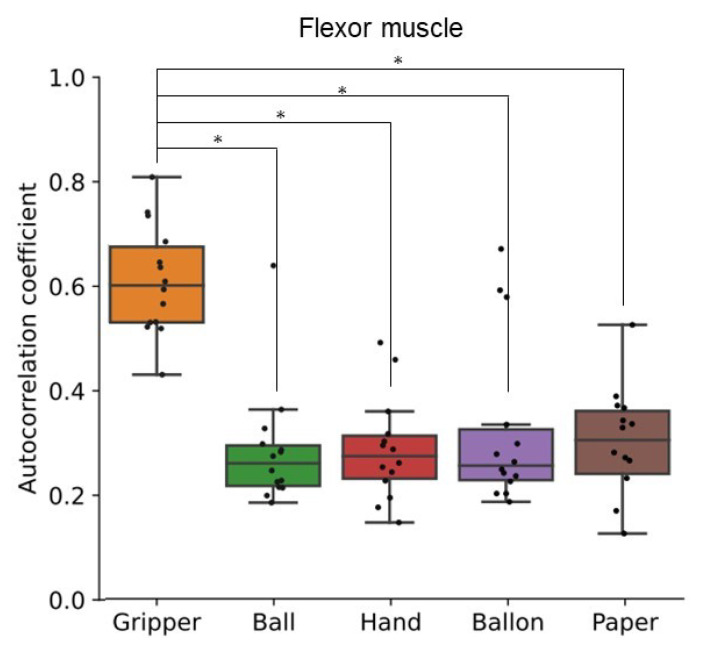
Local maxima in the absolute value of the autocorrelation coefficient of muscle deformation data for flexor muscles. Box plots of the maximum value of the local maxima in the absolute value of the autocorrelation coefficient for all participants’ flexor muscles. The training types were gripping-releasing of a gripper (gripper), holding of a ball (ball), palm clenching (hand), holding of a balloon (balloon), and crumpling of newspaper (paper). The symbol * means the significant difference.

**Figure 11:**
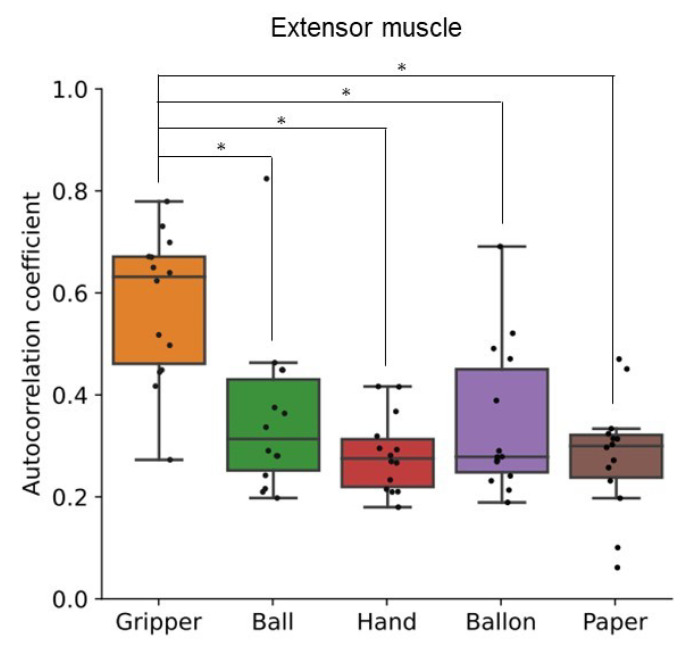
Local maxima in the absolute value of the autocorrelation coefficient of muscle deformation data for extensor muscles. Box plots of the maximum value of the local maxima in the absolute value of the autocorrelation coefficient for all participants’ extensor muscles. The training types were gripping-releasing of a gripper (gripper), holding of a ball (ball), palm clenching (hand), holding of a balloon (balloon), and crumpling of newspaper (paper). The symbol * means the significant difference.

Figure 9 shows autocorrelation coefficient results of flexor and extensor muscles for all participants. This figure is a correlogram, that is, the relationship between the autocorrelation coefficient value (vertical axis) and the shifted lag of the data (horizontal axis). The periodicity can be seen in the correlogram of the gripper task for all participants, although the frequency is different among participants. In the paper tasks, the periodicity can be seen periodicity for a few participants. In addition, Figure 10 and Figure 11 show a box plot of the maximum value of the local maxima in the absolute value of the autocorrelation coefficient for flexor and extensor muscles, respectively. The values in the gripper task were significantly higher than the other tasks. Autocorrelation of gripper task for both flexor and extensor muscles tended to be strong while autocorrelation of others tended to be slight. Consequently, only the gripper task could be detected as a periodic activity.

## 6. Discussion

The SD and autocorrelation coefficient derived by the proposed process with FirstVR (circular-array near-infrared sensor) could detect the variability and the periodicity of the applied load to the forearm muscles, respectively. The gripper task is periodic movement while the paper task is aperiodic movement. As shown in Figure 5 and Figure 6, the applied load to the forearm muscles in the paper task varied as well as in the gripper task. The gripper and paper exercises required a time change of activation for both flexor and extensor muscles. The isometric tasks did not require the variability of the muscle activity. The significance difference in the SD corresponds to the difference in variability of the training types. As shown in Figure 10 and Figure 11, the value of the local maxima in the absolute values of autocorrelation coefficient in the gripper task was significantly higher than in the other tasks. Gripping and releasing the gripper, that is spring-based resistance, is a periodic motion. On the other hand, the paper exercise task is aperiodic motion; the contraction speed and length of the muscles change randomly. Since the main distribution of the values in the paper task was less than approximately 0.35, the system showed that the applied load was aperiodic during the paper task. The autocorrelation coefficient index could identify whether the applied load was periodic fluctuation or not. Since the correlation coefficient between the peak value of the muscle deformation and peak number was just approximately 0.25, the effect of the physiological change, such as fatigue, might not be involved in the periodicity value for the gripper task.

As shown in Figure 7 and Figure 8, there were no significant differences among training types. The mean value in the ball task, which is the isometric hand-gripping task, tended to be higher for some participants. The load was applied to the flexor and extensor muscles in all conditions. Flexor muscles deformed more than extensor muscles, thus the means of deformation values of flexor muscles tended to be higher than extensor muscles. The mean value of the muscle deformation enables us to recognize whether the load was applied to the skeletal muscles or not.

The data of the maximum value of the local maxima in the absolute values of autocorrelation shows that the temporal activity patterns and rhythms of flexor and extensor muscles exhibit high reproducibility in the gripper task. On the contrary, in the paper exercise, there appears to be variability in the temporal activity patterns of flexor and extensor muscles. The paper task required the participants to finely control the hand-gripping force corresponding to the changeable state of the paper over time. As shown in Figure 9, we could find periodic patterns in the paper tasks for some participants. There was an individual difference in autocorrelation coefficient value. We assume that the randomness task requires the ability to move corresponding to the chaining state. Some participants might not be able to exert hand-gripping force corresponding to the changing paper resistance, and just outputted the patterned motion. We would investigate the relationship between the human condition or ability and motion randomness in the future.

OMG-based sensing of muscle activity demonstrated the feasibility of monitoring the applied load to the skeletal muscles for self-administered screening when repeating various training. The gold standard for estimating the applied load to skeletal muscles is the EMG-based method. Time-variability and periodical analysis for EMG was conducted for analyzing muscle fatigue [33,34,35]. The strength or power of the load can be estimated after rectification or integration of EMG signals [27,28]. EMG is affected directly by sweat due to a change in skin impedance. People might sweat during sports or training activities. In addition, attaching EMG sensors requires anatomical expertise. Circular-array near-infrared sensors can reduce the effect of sweat and make it easier to wear, which is an advantage for use in daily sports or training activity.

On the other hand, the way how the near-infrared light is reflected is complex. Some studies used near-infrared sensors to observe oxygenation (or deoxygenation), that is, the change in the blood involved in muscle contraction [44,45]. Several wavelengths of near-infrared lights are needed to estimate accurate muscle oxygenation (or deoxygenation), which requires the accurate attachment position of optical emitters and receivers above the target muscles. Considering oxygenation mechanism is related to EMG [40,49], oxygenation and muscle deformation, which is the change in path length of the light, are evoked simultaneously. Therefore, observation of change in reflectance of the near-infrared light, such as in [39], includes the effect of muscle deformation. In addition, it was reported that the reflectance of the near-infrared light also changes even with skin deformation [46]. Our study showed that muscle deformation can be estimated from one-wavelength of the near-infrared light. As shown in [42], the near-infrared-light receiver observed the reflectance outputted by a closer near-infrared-light emitter. We consider that this study extracted muscle deformation elements by deriving the mean values of the region close to flexor or extensor muscles after calibration. Relative change elicited by muscle deformation from a initial state became visible thanks to calibration, and variable and aperiodical elements of time-series data of muscle deformation could be observed. In addition, the derivation of means of multiple channels compensates for the failure of sensing from some channels and might enable observation of time-variability and periodicity of the applied load.

The are some limitations in this study. First, the appropriate positional relationship between the channels and muscles is still unclear, and thus it is difficult to observe the variability and periodicity of deformation of each muscle in the forearm in more detail. Redundant information is needed for observation. Independent component analysis would be helpful for the analysis of each muscle deformation in future work. In addition, adaptive adjustment of sensing position and processing to individual differences is also limited. In particular, maintaining the spatial relationship between the sensing channels and muscle anatomical position is difficult in case of large arm movement. The synergy of muscles, especially the relationship between flexors (ventral side) and extensors [50], might also have an effect on monitoring.

## 7. Conclusions

Hand-gripping is one of the fundamental functions of human physical activity. Bernstein’s idea of “repetition without repetition” suggests that motor control function should be trained under changing states. The randomness level of load should be visualized for self-administered screening when repeating various training tasks under changing states. The aim of this study is to develop a sensing methodology of random loads applied to both the agonist and antagonist skeletal muscles while performing physical tasks. In the experiment, 14 participants conducted the gripping tasks with a gripper, ball, balloon, palm clenching, and paper. Crumpling pieces of paper (paper exercise) involves randomness because the resistance force of the paper changes depending on the shape and layers of the paper. OMG during gripping tasks was measured, and time-series features were analyzed. The gripper and paper tasks both involve repetitive movements of finger flexion and extension. On the other hand, ball, palm clenching, and balloon primarily entail sustained isometric contractions, exhibiting significant differences in muscle activity and contraction states. The OMG-based measuring could detect the feature of the muscle movements during isometric (not varying) and isokinetic training (varying) by reading the SDs of time-series muscle deformation data. The mean value of the muscle deformation enables us to recognize whether the load was applied to the skeletal muscles or not. Consequently, it was found that the paper exercise task could apply randomness load to both the agonist and antagonist skeletal muscles for gripping. Our system could detect the random movement of muscles during training.

This study is the first examination to detect time-variability and periodicity/aperiodicity in data on skeletal muscle deformation using a circular array of near-infrared lights. Using OMG might help us conduct the training more easily and comfortably in daily life compared to EMG and FMG. We would conduct the test of training effect to monitor and analyze the time-series muscle deformation using OMG during hand-gripping training. In the future, we would develop a more variety of examination methods during both training and exercise based on OMG. In addition, independent component analysis of the sensing channels would be made to observe each muscle in more detail. Moreover, a method of maintaining the spatial relationship between sensing channel and muscle anatomical position for each individual in case of large arm movements would be considered. Furthermore, the effect of the synergy of muscles, especially the relationship between flexors (ventral side) and extensors, would be investigated.

## Data Availability

The data used to support the findings of this study are available from the corresponding author upon request.
